# Patients with Rheumatoid Arthritis and Chronic Pain Display Enhanced Alpha Power Density at Rest

**DOI:** 10.3389/fnhum.2016.00395

**Published:** 2016-08-04

**Authors:** Francisco M. Meneses, Fernanda C. Queirós, Pedro Montoya, José G. V. Miranda, Selena M. Dubois-Mendes, Katia N. Sá, Cleber Luz-Santos, Abrahão F. Baptista

**Affiliations:** ^1^Graduate Program in Medicine and Health, School of Medicine, Federal University of BahiaSalvador, Brazil; ^2^Functional Electrostimulation Laboratory, Biomorphology Department, Health Sciences Institute, Federal University of BahiaSalvador, Brazil; ^3^Department of Psychology, Research Institute of Health Sciences, University of Balearic IslandsPalma de Mallorca, Spain; ^4^Nucleus of Innovation and Technology in Rehabilitation, Institute of Physics, Federal University of BahiaSalvador, Brazil; ^5^Physiotherapy Program, Bahia School of Medicine and Public HealthSalvador, Brazil

**Keywords:** rheumatoid arthritis, chronic pain, EEG, delta rhythm, theta rhythm, alpha rhythm, beta rhythm

## Abstract

Patients with chronic pain due to neuropathy or musculoskeletal injury frequently exhibit reduced alpha and increased theta power densities. However, little is known about electrical brain activity and chronic pain in patients with rheumatoid arthritis (RA). For this purpose, we evaluated power densities of spontaneous electroencephalogram (EEG) band frequencies (delta, theta, alpha, and beta) in females with persistent pain due to RA. This was a cross-sectional study of 21 participants with RA and 21 healthy controls (mean age = 47.20; SD = 10.40). EEG was recorded at rest over 5 min with participant's eyes closed. Twenty electrodes were placed over five brain regions (frontal, central, parietal, temporal, and occipital). Significant differences were observed in depression and anxiety with higher scores in RA participants than healthy controls (*p* = 0.002). Participants with RA exhibited increased average absolute alpha power density in all brain regions when compared to controls [*F*_(1.39)_ = 6.39, *p* = 0.016], as well as increased average relative alpha power density [*F*_(1.39)_ = 5.82, *p* = 0.021] in all regions, except the frontal region, controlling for depression/anxiety. Absolute theta power density also increased in the frontal, central, and parietal regions for participants with RA when compared to controls [*F*_(1, 39)_ = 4.51, *p* = 0.040], controlling for depression/anxiety. Differences were not exhibited on beta and delta absolute and relative power densities. The diffuse increased alpha may suggest a possible neurogenic mechanism for chronic pain in individuals with RA.

## Introduction

Rheumatoid arthritis (RA) is a chronic, autoimmune disease of unknown etiology (Firestein, 2003). A recent systematic literature review estimated the global prevalence to be 0.24% (95% CI: 0.23–0.25%; Cross et al., 2014). Gender plays an important role, as women are twice as likely to present the condition (mean 0.35%; 95% CI: 0.34–0.37) than males (mean 0.13%; 95% CI: 0.12–0.13; Mikkelsen et al., 1967).

RA is characterized by peripheral and symmetric polyarthritis, affecting the synovial membranes of joints, leading to pain, and joint deformities (McInnes and Schett, 2011). RA was recently associated with neuropathic pain (Mendes et al., 2014; Walsh and McWilliams, 2014; Koop et al., 2015) which may be present, among other factors, because of entrapment neuropathies, the use of certain drugs and central sensitization. Pain is perhaps the most common symptom and the most related to disability in RA patients (Skevington, 1986; Firestein, 2003; Walsh and McWilliams, 2014). However, the quantification and characterization of pain is a challenge for clinicians, since the experience of pain is individual and subjective (Pimenta and Teixeira, 1996; de Vries et al., 2013). Scales and questionnaires have been used in clinical practice to describe pain intensity, as well as its temporal and qualitative aspects.

The electroencephalogram (EEG) is a promising tool for pain evaluation in clinical settings (Jones et al., 2012), since it can provide useful information about the central mechanisms involved in the maintenance of chronic pain in rheumatic diseases (Lee et al., 2011). In general, the assessment of EEG characteristics during wakefulness demonstrated that chronic neuropathic pain usually is associated with EEG slowing, increased power density and peak frequency in the low frequency ranges (theta, alpha; Boord et al., 2008; Olesen et al., 2011; de Vries et al., 2013; Jensen et al., 2013; van den Broeke et al., 2013). Several authors have further argued that EEG abnormalities in chronic pain could be due to a dysfunction of top-down or bottom-up thalamic modulation (thalamocortical dysrhythmia; Llinás et al., 1999, 2005; Sarnthein et al., 2006). Moreover, the fact that patients with chronic low back pain did not show a similar pattern of EEG slowing seems to raise the question of whether this could be a relevant marker for distinguishing between the neuropathic and the nociceptive nature of pain (Schmidt et al., 2012).

RA is fundamentally an inflammatory disease, associated with severe and disabling pain. Although inflammation of joints and other musculoskeletal tissues are the main sources of nociceptive pain in RA (Schaible et al., 2002; Schaible, 2014), recent studies have identified neuropathic pain components within the symptoms of this disease (Ahmed et al., 2014; Mendes et al., 2014; Koop et al., 2015). One of the main candidates to explain the presence of neuropathic pain symptoms is central sensitization (Ahmed et al., 2014). This condition is a consequence of pathological enhancement in nociceptive neuronal function due to maintained nociceptive transmission or decreased endogenous inhibition (Latremoliere and Woolf, 2009). Central sensitization *per se* is associated to the development of neuropathic pain complaints (Mease et al., 2011), which has been identified in patients with RA (Meeus et al., 2012). This maladaptive condition of the central nervous system may be related to spreading of symptoms, decreased pain thresholds, and the poor relation between disease activity and symptoms in RA (Atzeni et al., 2011; Meeus et al., 2012; Hochman et al., 2013). Furthermore, these modifications in the processing of pain at the central level have already been characterized by somatosensory EEG event-related potentials (Wendler et al., 2001), but not by EEG activity at rest.

Given the combination of nociceptive and neuropathic pain in RA, the investigation of quantitative EEG at rest may shed light into its pathophysiology. It may also reveal whether signs of thalamocortical dysrhythmia are present. Therefore, the objectives of the current study were two-fold: (a) to compare EEG activity at rest in patients with RA to healthy controls, and (b) to evaluate the relationship between pain characteristics and EEG activity in patients with RA.

## Materials and methods

### Participants

Twenty-one women with RA (mean = 47.92, SD = 12.36) and 21 healthy controls (HC; mean = 46.41, SD = 8.30) participated in this cross-sectional study and assessed between August 2013 and October 2014. The participants with RA were recruited from a third-party reference center in Bahia (Brazil) and had received a diagnosis from a rheumatologist, conforming with the criteria from the American College of Rheumatology (Aletaha et al., 2010). Patients were included if they were suffering from chronic pain (pain lasting more than 6 months), during more than 3 days per week, and predominantly located in the joints associated or not with deformities and/or joint range of motion. Participants were excluded if they were diagnosed with any other rheumatologic disease in addition to RA, or reported the use of centrally acting substances. The control group did not report chronic pain and was pain-free on the day of the experiment. Three milliliters of venous blood were collected from the participants with RA in order to analyze the Erythrocyte Sedimentation Rate and C-reactive protein.

Table 1 presents sociodemographic and clinical characteristics for the entire sample and the two groups, as well as the results of tests comparing averages and proportions for the two groups. Significant differences between RA patients HC only appeared in anxiety/depression scores.

**Table 1:** **Comparison of demographic, behavioral, and clinical characteristics of women with Rheumatoid Arthritis and Healthy Controls**.

	**Total sample (*n* = 42) *N* (%) or average (SD)**	**Healthy controls (*n* = 21) *N* (%) or average (SD)**	**RA Patients (*n* = 21) *N* (%) or average (SD)**	***p*-value**
**DEMOGRAPHIC CHARACTERISTICS**				
Age, in years	47.16 (10.42)	46.41(8.30)	47.92 (12.36)	0.64
Variation (min - max)	–	33–59	23–69	
Level of education				0.08
HS incomplete	11 (26.19)	4 (19.05)	7 (33.33)	
Completed HS – some College	20 (47.62)	8 (38.10)	12 (57.14)	
College or higher	11 (26.19)	9 (42.86)	2 (9.52)	
Race/color				0.54
White	3 (7.14)	2 (9.52)	1 (4.76)	
Black/Afro-Brazilian	21 (50)	9 (42.86)	12 (57,14)	
Mixed race	13 (30.95)	6 (28.57)	7 (33.33)	
Others^a^	5 (11.90)	4 (19.05)	1 (4.76)	
Marital Status				0.31
Single	11 (26.19)	3 (14.29)	8 (38.1)	
Married/Live with partner	20 (47.62)	12 (57.14)	8 (38.1)	
Separated/Divorced/ Widow(er)	11 (26.19)	6 (28.57)	5 (23.81)	
**HEALTH BEHAVIOR**				
Smoking				0.76
No	31 (73.81)	16 (76.19)	15 (71.43)	
Yes	8 (19.05)	3 (14.29)	5 (23.81)	
Former smoker	3 (7.14)	2 (9.52)	1 (4.76)	
Alcohol consumption				0.06
Never	23 (54.76)	8 (38.10)	15 (71.43)	
Occasionally^b^	19 (45.24)	13 (61.90)	6 (28.57)	
Physical activity				0.31
Sedentary	20 (47.62)	8 (38.10)	12(57.14)	
Occasionally	9 (21.43)	4(19.05)	5(23.81)	
Moderate/intense	13 (30.95)	9(42.86)	4(19.05)	
**CLINICAL CHARACTERISTICS**				
Diabetes Mellitus	3 (7.14)	2 (9.52)	1 (4.76)	1.00
Thyroid problem	10 (23.81)	4 (19.05)	6 (28.57)	0.72
Laterality in upper limb				1.00
Right	39 (92.86)	20 (95.24)	19 (90.48)	
Left	3 (7.14)	1 (4.76)	2 (9.52)	
Depression/Anxiety (HADS)				0.002^*^
With anxiety and/or depression	19 (45.24)	4 (19.05)	15 (71.43)	

*Differences were tested for continuous variables between the groups using the Student's t-test and for categories using Fisher's exact test*.

* *Significant at level 0.05*.

*SD, Standard Deviation; RA, Rheumatoid Arthritis; HS, High School; HADS, Hospital Anxiety and Depression Scale*.

a Others, the sum of individuals auto-declared “Yellow/Oriental” and “Red/Indian.”

b *Occasionally, weekends without any incidents of drunkenness. The original categories in this variable also included the following options: occasionally with incidents of drunkenness; frequently without any incidents of drunkenness and frequently with incidents of drunkenness. None of the participants selected these options*.

The average duration of the disease in the group of participants with RA was 107.4 ± 45.9 months, with an average medical follow-up time of 93.4 ± 43.4 months. The medications most frequently used by these participants were Metotrexate (52.4%), Infliximab (19%), and Prednisone (38.1%). Most of the RA patients reported high (*n* = 8) or moderate (*n* = 10) disease activity. Only two patients were in remission and one presented low disease activity. The main neuropathic pain descriptors (DN4 questionnaire) were numbness (71.4%), tingling (61.9%), and electric shock (57.1%). A total of 57.1% of the RA participants reported neuropathic pain, according to the DN4. The clinical pain characteristics of RA participants are described in detail in Table 2.

**Table 2:** **Characteristics of the pain in patients with Rheumatoid Arthritis**.

**CHARACTERISTICS OF THE PAIN**	
Disease activity^a^	***N*** **(%)**
Remission	2 (9.52)
Low	1 (4.76)
Moderate	10 (47.62)
High	8 (38.0)
Neuropathic pain^a^	***N*** **(%)**
With neuropathic pain	12 (57.14)
Without neuropathic pain	9 (42.86)
	**Average (*****SD*****)**
Number of pain descriptors (McGill)	13.57 (5.90)
McGill pain index	28.14 (13.37)

*SD, Standard Deviation*.

a *Evaluated using the Douleur Neuropathique 4 (DN4) questionnaire, with a variation of between 0 and 10 and average of 4.10 (DP = 2.51). Patients with neuropathic pain were those with a score equal to or higher than 4.0*.

*Number of pain descriptors and McGill pain index: measured using the McGill Scale (1996)*.

Participants were verbally informed about the details of the study and all questions answered at the time of recruitment. After agreeing to participate, a written consent was obtained and a printed copy was provided to subjects. The study was conducted in compliance with the principles of the Declaration of Helsinki, and was approved by the Research Ethics Committee at the *Escola Bahiana de Medicina e Saúde Pública* (Bahia School of Medicine and Public Health; reference #1395/2011).

### Psychological questionnaires

All participants underwent a semi-standardized interview, including socio-demographic data (age, marital status, level of education, alcohol consumption, smoking, and practice of physical activities) and assessment of mood through the Hospital Anxiety and Depression Scale (HADS). The HADS is a 14-items questionnaire for the assessment of anxiety and depression symptoms (Bjelland et al., 2002). The maximum score on the depression subscale is 21, with a cut-off point at nine. The maximum score on the anxiety subscale is also 21, with a cut-off point at seven. We used an adapted and validated Brazilian version of this scale (Castro et al., 2006).

The following questionnaires were completed by participants with RA only:

*McGill Pain Questionnaire*. This questionnaire evaluates the subjective and multidimensional experience of pain, providing quantitative measures of clinical pain. It comprises the following categories of pain descriptors: sensitive-discriminative; affective-motivational; cognitive-evaluative; and miscellaneous (Melzack, 1975). In the present study, we used an adapted and validated Brazilian version of this questionnaire (Pimenta and Teixeira, 1996). The maximum “number of pain descriptors” is 20. The “total pain level” was defined as the sum of values for pain intensity, with a maximum score of 78.
*The Neuropathic Pain Diagnostic Questionnaire (Douleur Neuropathique 4 - DN4)*. This 10-item questionnaire was designed to assess neuropathic pain and includes pain descriptors (7 items) and a bedside examination (3 items; Bouhassira et al., 2005). The final score falls within a scale from 0 to 10. Scores higher than three indicate the presence of neuropathic pain. We used a validated Brazilian version of this questionnaire (Santos et al., 2010).
*Disease Activity Score in 28 Joints (DAS28)*. The goal of the questionnaire is to evaluate the level of disease activity in RA patients (Prevoo et al., 1995). It assesses 28 joints (shoulders, elbows, wrists, proximal interphalangeal and metacarpophalangeal, and knees, bilaterally), counting the number of painful joints without considering pain intensity. A joint is considered “painful” if some level of discomfort is present, even if the pain is not intense. The total score varies from 0 to 10. Activity level was classified according to the following cut-off points: remission ≤ 2.6; low ≤ 3.2; moderate ≤ 5.1; and high activity > 5.1 (Pinheiro, 2007).

### EEG recording

EEG data were recorded using a standard amplifier (BRAIN NET 36, EMSA Brazil) from 20 electrodes and two references located on the auricular region (A1 and A2). Active EEG electrodes were placed according to the international 10–20 system at following locations: F7, T3, T5, Fp1, F3, C3, P3, O1, F8, T4, T6, Fp2, F4, C4, P4, O1, Fz, Cz, Pz, and Oz. The sampling rate was 200 Hz and a ground electrode was placed in the frontal region (Fpz). Electrode impedance was kept below 5 kΩ. Participants were instructed to stay relaxed with eyes closed but were monitored so that they were awake throughout the 5 min recording.

The EEG data were analyzed by using the EEGLAB software (version 13). The signals were filtered with a band-pass filter between 0.5 and 50 Hz. Continuous EEG data were segmented in epochs of 1.28 s, which allowed a consistent evaluation of power densities in the frequency range of 1.5–30 Hz. A semi-automated rejection protocol was used to remove artifacts, with an upper limit of 1000 μV and a lower limit of −1000 μV. After the artifact rejection protocol, a minimum of 170 epochs were kept for each participant, an equivalent to roughly 3.5 min. Since we had decided to analyze 2 min for each participant, we selected the central epochs in order to standardize the selection process and avoid selection bias. Thus, only data between epochs 50 and 142 (93 epochs, nearly 2 min) of the EEG recording were analyzed.

Power spectra were calculated by applying a fast Fourier transform for each epoch. Power densities of each epoch and electrode were averaged separately for each participant. The average power densities were grouped into delta [1.5–3.5 Hz], theta [4–7 Hz], alpha [8–12 Hz], and beta [13–30 Hz] frequency bands. In addition, regions of interest (ROI) were computed by averaging power densities at the four frequency bands for the following groups of electrodes: frontal (Fp1, Fp2, F3, Fz, F4), central (C3, Cz, C4), parietal (P3, Pz, P4), occipital (O1, Oz, O2), and temporal (T3, T5, T4, T6). After processing data for absolute power densities, the same was done for relative power densities. These were computed dividing electrode's values in each one of the analyzed frequencies by their values in the total power spectrum. The results for relative power density were also analyzed and displayed by the same ROIs.

### Statistical analysis

Data from the questionnaires were analyzed by using Student *t*-tests to examine differences between the two groups. The differences on the categorical variables were analyzed by using Fisher's Exact Test, as cells with a frequency equal to or less than five were observed in the bivariate analyses. After confirming normality of the data by using Shapiro–Wilk test and Q-Q plots, differences in absolute and relative EEG power densities between the groups was analyzed by using repeated-measures ANOVA with the factors “group” and “region” (ROI) after controlling for anxiety/depression symptoms. Violations of sphericity assumption were corrected by using Greenhouse-Geisser epsilons.

Finally, Pearson zero-order correlations were computed between mean power densities and pain variables (disease activity, neuropathic/nociceptive pain, and McGill outcome variables). All pain variables were normally distributed (using Kolmogorov–Smirnov test). A *p*-value of 5% was used to accept statistically significant differences between the two groups. The *p*-value was corrected for multiple comparisons using the Bonferroni method when necessary. The SPSS 20.0 software package was used for all analyses.

## Results

### Difference between groups on EEG absolute power density

**Figure 1:**
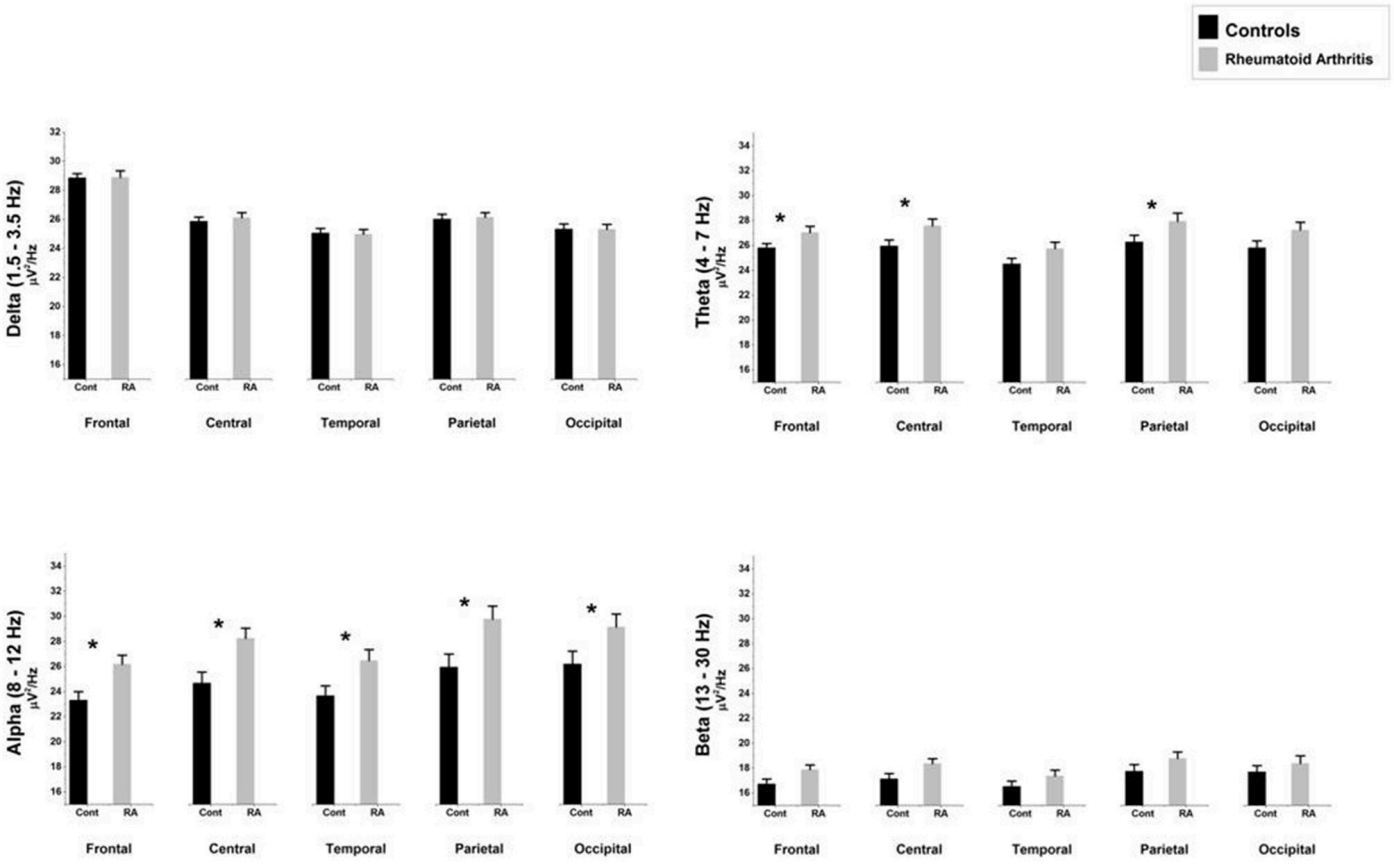
**Comparison of absolute mean power densities (μ V^**2**^/Hz) for delta, theta, alpha, and beta bands between patients with rheumatoid arthritis and healthy controls in five brain regions**. An asterisk indicates significant statistical difference (*p* < 0.05).

An ANOVA looking at the full power spectrum (1.5–30 Hz) yielded a significant effect of “group” [*F*_(1, 39)_ = 5.12, *p* = 0.029], indicating that patients displayed overall higher power density than HC. We also found a significant “region” effect [*F*_(4, 156)_ = 18.27, *p* < 0.0.0000001, epsilon GG = 0.595]. Although there were non-significant differences due to the interaction between group and region [*F*_(4, 156)_ = 0.51, *p* = 0.605, epsilon GG = 0.505], mean comparisons in the *post-hoc* analysis (using Bonferroni correction to adjust for multiple comparisons) revealed that RA patients displayed higher power density than HC at frontal (mean difference = 1.589, *p* = 0.026), central (mean difference = 2.006, *p* = 0.015), temporal (mean difference = 1.772, *p* = 0.040), and parietal (mean difference = 2.178, *p* = 0.038) electrodes. After observing this difference in the full power spectrum, we proceeded to look at frequencies of interest. Our discussion will focus on the four frequency bands reported below. Table 3 and Figure 1 display the average absolute power density values for the analyzed EEG frequency bands (delta, theta, alpha, and beta) across the five ROIs.

**Table 3:** **Average absolute power density values by regions of interest, controlling for symptoms of anxiety/depression**.

**Frequency bands (Regions of Interest)**	**Controls (*n* = 21)**	**RA Patients (*n* = 21)**	***F*_(1.39)_**	***P*-value**
**Delta (1.5–3.5 Hz)**			2.363	0.132
Frontal	28.86 (1.29)	28.88 (2.09)		
Central	27.79 (1.50)	28.07 (1.88)		
Temporal	26.82 (1.75)	26.74 (1.70)		
Parietal	27.96 (1.82)	28.10 (1.76)		
Occipital	27.16 (1.86)	27.14 (1.78)		
**Theta (4–7 Hz)**			4.505	0.040^*^
Frontal	25.82 (1.54)	27.03 (2.31)		
Central	25.96 (2.16)	27.57 (2.55)		
Temporal	24.51 (2.13)	25.73 (2.41)		
Parietal	26.28 (2.42)	27.93 (2.98)		
Occipital	25.80 (2.54)	27.23 (2.89)		
**Alpha (8–12 Hz)**			6.385	0.016^*^
Frontal	23.31 (3.17)	26.18 (3.30)		
Central	24.68 (4.01)	28.24 (3.76)		
Temporal	23.67 (3.68)	26.47 (4.07)		
Parietal	25.96 (4.72)	29.78 (4.80)		
Occipital	26.20 (4.72)	29.14 (4.93)		
**Beta (13–30 Hz)**			3.352	0.075
Frontal	16.73 (1.80)	17.87 (1.79)		
Central	17.14 (1.94)	18.36 (1.76)		
Temporal	16.53 (1.97)	17.37 (2.12)		
Parietal	17.74 (2.45)	18.77 (2.35)		
Occipital	17.69 (2.36)	18.38 (2.77)		

* *Significant at level 0.05. ANOVA of repeated measures*.

#### Delta (1.5–3.5 Hz)

The ANOVA yielded only a significant effect due to “region” [*F*_(4, 156)_ = 15.39, *p* < 0.0000001, epsilon GG = 0.640]. No significant effects of “group” [*F*_(1, 39)_ = 2.36, *p* = 0.132] or the interaction between “group” and “region” [*F*_(4, 156)_ = 0.535, *p* = 0.631, epsilon GG = 0.470] were observed on absolute delta power densities.

#### Theta (4–7 Hz)

The ANOVA yielded a significant effect of “group” [*F*_(1, 39)_ = 4.51, *p* = 0.040], indicating that patients displayed higher absolute theta power density than HC. We also found a significant “region” effect [*F*_(4, 156)_ = 18.22, *p* < 0.0000001, epsilon GG = 0.634], showing that highest power densities were found at parietal, and central electrodes, whereas the lowest ones appeared at frontal electrodes. Although there were non-significant differences due to the interaction between group and region [*F*_(4, 156)_ = 0.71, *p* = 0.526, epsilon GG = 0.634], mean comparisons in the *post-hoc* analysis (using Bonferroni correction to adjust for multiple comparisons) revealed that RA patients displayed higher absolute theta power density than HC at frontal (mean difference = 1.530, *p* = 0.039), central (mean difference = 2.023, *p* = 0.024), and parietal (mean difference = 2.067, *p* = 0.043) electrodes.

#### Alpha (8–12 Hz)

The ANOVA yielded a significant effect of “group” [*F*_(1, 39)_ = 6.39, *p* = 0.016], indicating that patients displayed higher absolute alpha power density than controls. We also found a significant “region” effect [*F*_(4, 156)_ = 37.222, *p* < 0.0000001, epsilon GG = 0.480] showing that highest power densities were found at parietal, occipital, and central electrodes, whereas the lowest ones appeared at frontal and temporal electrodes. Although there were non-significant differences due to the interaction between group and region [*F*_(4, 156)_ = 1.69, *p* = 0.192, epsilon GG = 0.480], mean comparisons in the *post-hoc* analysis (using Bonferroni correction to adjust for multiple comparisons) revealed that RA patients displayed higher absolute alpha power density than HC at all five ROIs: frontal (mean difference = 3.019, *p* = 0.015), central (mean difference = 3963, *p* = 0.008), temporal (mean difference = 3.317, *p* = 0.025), parietal (mean difference = 4.437, *p* = 0.015), and occipital (mean difference = 3783, *p* = 0.035).

#### Beta (13–30 Hz)

The ANOVA yielded only a significant effect due to “region” [*F*_(4, 156)_ = 8.97, *p* = 0.000207, epsilon GG = 0.538]. No significant effects of “group” [*F*_(1, 39)_ = 3.35, *p* = 0.075] or the interaction between “group” and “region” [*F*_(4, 156)_ = 0.12, *p* = 0.997, epsilon GG = 0.538] were observed on absolute beta power densities.

### Difference between groups on EEG relative power density

**Figure 2:**
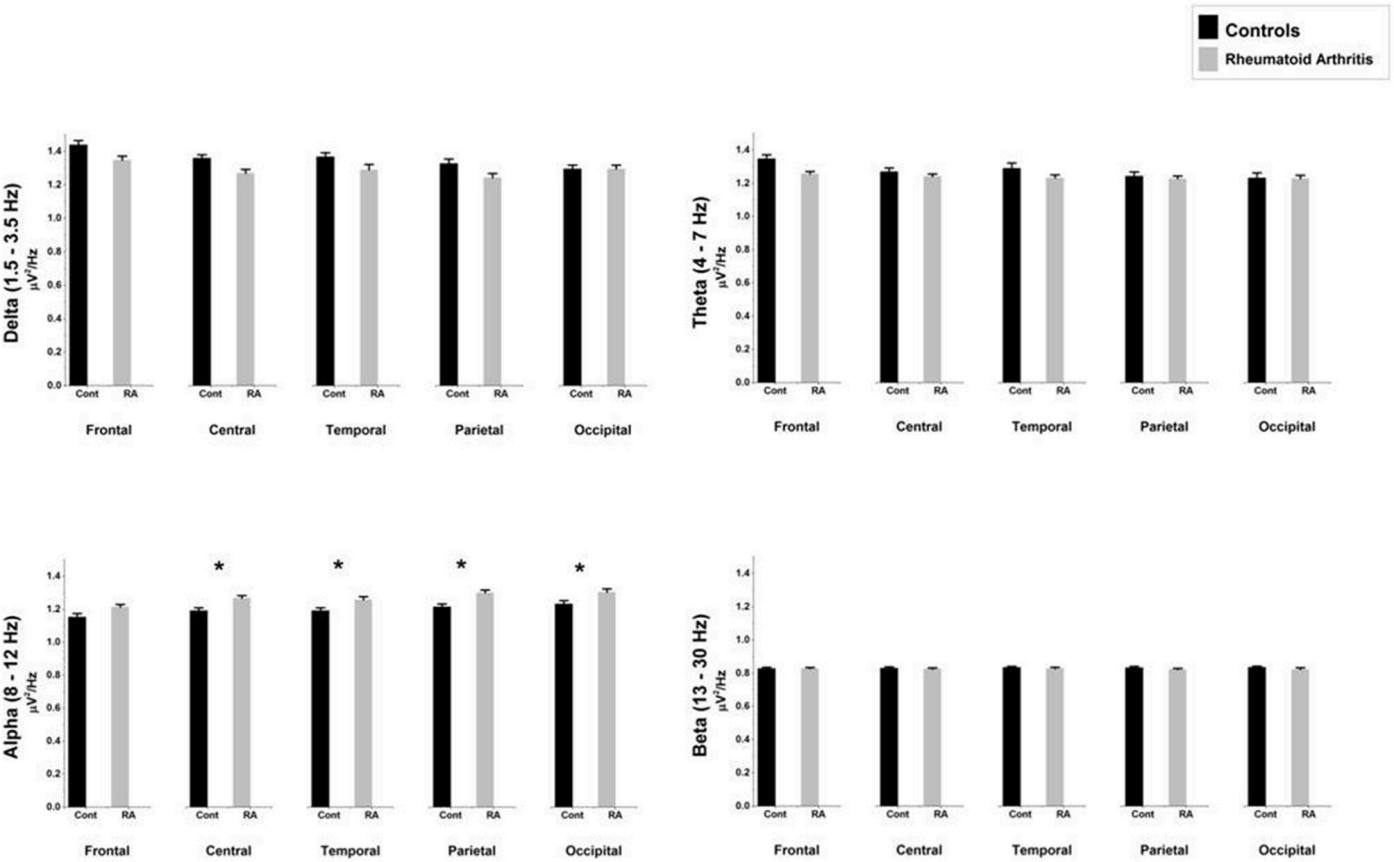
**Comparison of relative mean power densities (μ V^**2**^/Hz) for delta, theta, alpha, and beta bands between patients with rheumatoid arthritis and healthy controls in five brain regions**. An asterisk indicates significant statistical difference (*p* < 0.05).

Table 4 and Figure 2 show the average relative power density values for the analyzed EEG frequency bands (delta, theta, alpha, and beta) across the five ROIs.

**Table 4:** **Average relative power density values by regions of interest, controlling for symptoms of anxiety/depression**.

**Frequency bands (Regions of Interest)**	**Controls (*n* = 21)**	**RA Patients (*n* = 21)**	***F*_(1.39)_**	***P*-value**
**Delta (1.5–3.5 Hz)**			3.159	0.083
Frontal	1.44 (0.12)	1.35 (0.11)		
Central	1.36 (0.09)	1.27 (0.11)		
Temporal	1.37 (0.11)	1.29 (0.15)		
Parietal	1.33 (0.12)	1.24 (0.12)		
Occipital	1.30 (0.11)	1.23 (0.14)		
**Theta (4–7 Hz)**			0.565	0.457
Frontal	1.28 (0.07)	1.26 (0.07)		
Central	1.27 (0.07)	1.24 (0.07)		
Temporal	1.24 (0.07)	1.23 (0.08)		
Parietal	1.24 (0.08)	1.23 (0.08)		
Occipital	1.23 (0.07)	1.23 (0.09)		
**Alpha (8–12 Hz)**			5.823	0.021^*^
Frontal	1.15 (0.10)	1.21 (0.08)		
Central	1.19 (0.10)	1.27 (0.08)		
Temporal	1.19 (0.08)	1.26 (0.08)		
Parietal	1.21 (0.09)	1.30 (0.08)		
Occipital	1.23 (0.09)	1.30 (0.10)		
**Beta (13–30 Hz)**			0.440	0.511
Frontal	0.83 (0.04)	0.83 (0.03)		
Central	0.83 (0.04)	0.83 (0.03)		
Temporal	0.83 (0.03)	0.83 (0.04)		
Parietal	0.83 (0.03)	0.82 (0.03)		
Occipital	0.84 (0.03)	0.82 (0.05)		

* *Significant at level 0.05. ANOVA of repeated measures*.

#### Delta (1.5–3.5 Hz)

The ANOVA yielded only a significant effect due to “region” [*F*_(4, 156)_ = 22.89, *p* < 0.0000001, epsilon GG = 0.582]. No significant effects of “group” [*F*_(1, 39)_ = 3.16, *p* = 0.083] or the interaction between “group” and “region” [*F*_(4, 156)_ = 0.021, *p* = 987, epsilon GG = 0.582] were observed on relative delta power densities.

#### Theta (4–7 Hz)

The ANOVA yielded only a significant effect due to “region” [*F*_(4, 156)_ = 12.63, *p* = 0.000041, epsilon GG = 0.554]. No significant effects of “group” [*F*_(1, 39)_ = 0.56, *p* = 0.457] or the interaction between “group” and “region” [*F*_(4, 156)_ = 0.053, *p* = 960, epsilon GG = 0.554] were observed on relative theta power densities.

#### Alpha (8–12 Hz)

The ANOVA yielded a significant effect of “group” [*F*_(1, 39)_ = 5.82, *p* = 0.021], indicating that patients displayed higher relative alpha power density than controls. We also found a significant “region” effect [*F*_(4, 156)_ = 23.09, *p* < 0.0000001, epsilon GG = 0.613] showing that highest relative power densities were found at parietal and occipital electrodes, whereas the lowest ones appeared at central and temporal electrodes. Although there were non-significant differences due to the interaction between group and region [*F*_(4, 156)_ = 0.83, *p* = 0.46, epsilon GG = 0.613], mean comparisons in the *post-hoc* analysis (using Bonferroni correction to adjust for multiple comparisons) revealed that RA patients displayed higher relative alpha power density than HC at four ROIs: central (mean difference = 0.077, *p* = 0.020), temporal (mean difference = 0.064, *p* = 0.039), parietal (mean difference = 0.087, *p* = 0.008), and occipital (mean difference = 0.075, *p* = 0.034).

#### Beta (13–30 Hz)

The ANOVA yielded no significant effect due to “region” [*F*_(4, 156)_ = 1.22, *p* = 0.304, epsilon GG = 0.580], “group” [*F*_(1, 39)_ = 0.44, *p* = 0.511] or the interaction between “group” and “region” [*F*_(4, 156)_ = 0.26, *p* = 0.803, epsilon GG = 0.580] were observed on relative beta power densities.

### Relationship between pain characteristics and absolute and relative EEG activity

Correcting for multiple comparisons using Bonferroni method, none of the Pearson correlations between pain characteristics and power density in the delta, theta, alpha, and beta EEG frequency bands were significant.

## Discussion

This study showed that participants with RA and chronic pain presented higher theta and alpha absolute power densities at rest in comparison to healthy individuals, whereas no group differences were found for absolute power density of the beta and delta EEG band. When looking at relative power densities, we only found group differences in the alpha band.

Our most consistent finding was in the alpha frequency, which was increased among participants with RA for both absolute and relative power densities. We progressed from absolute to relative power analysis because there was an increase in the total spectrum power density for the RA group. If we solely assessed absolute power density, as have the majority of large studies in this area (Pinheiro et al., 2016), we would not be able to state that there were specific differences between groups, since these differences could be related to the general increase in the total spectrum power density.

The increased alpha band power density in RA participants seems to be associated with specific pathological characteristics of the disease. Earlier studies have shown similar results in conditions of mental fatigue (Tran et al., 2014) and emotional stress (Vanneste et al., 2014), which are characteristic symptoms of patients with RA. In this sense, increased alpha power density has already been shown in individuals with tinnitus (Vanneste et al., 2014). Moreover, Sarnthein and Jeanmonod found increased spectral power density in the lower alpha range (7–9 Hz) in all cerebral regions in patients with neurogenic pain (Sarnthein and Jeanmonod, 2008). Similar results have also been evident in individuals with neuropathic pain due to spinal cord injury (Jensen et al., 2013), chronic pancreatitis (Drewes et al., 2008), and breast cancer (van den Broeke et al., 2013).

It is possible that the constant awareness in the expectation of pain may play a role in the increase of alpha power at rest (Babiloni et al., 2008, 2010). Previous studies have already shown that pain expectation activates the pain network, including “emotional” areas (Sawamoto et al., 2000; Koyama et al., 2005), and modulates alpha activity (Franciotti et al., 2009). However, the majority of studies that investigated the association between alpha related synchronization/desynchronization and pain used experimental paradigms (Peng et al., 2015). A recent review (Pinheiro et al., 2016) showed that alpha power may be increased in the resting state EEG, but the mechanisms for such increase still need to be investigated in depth. We did find group differences on depression/anxiety, leading us to control for these variables in the ANOVAs with the EEG data. Thus, we feel that the increase on alpha frequency in RA participants as compared to healthy controls was not influenced by participant's high levels of anxiety/depression.

Our results also revealed an increase in absolute theta power density in the participants with RA. This finding cannot be considered specific because of the increase in total spectral power seen in this group. However, the findings are in agreement with previous studies, showing increased theta power density in patients with migraine, fibromyalgia, neuropathic pain, and chronic pain secondary to low back pain (Sarnthein et al., 2006; Stern et al., 2006; Bjørk et al., 2009; Jensen et al., 2013; Vuckovic et al., 2014). Thalamic dysregulations such as thalamocortical dysrhythmia (TCD) have been described in individuals with neuropathic pain and could possibly explain our findings of enhanced absolute theta power density in RA patients (Sarnthein et al., 2006; Walton and Llinas, 2010; de Vries et al., 2013). Previous studies have shown that increased theta power density in patients with chronic neuropathic pain may be related to thalamic disinhibition due to decreased top-down or bottom-up modulation (Llinás et al., 1999, 2005; Sarnthein et al., 2006). In this sense, Stern et al. described increased theta power density in multiple areas of the pain matrix, including parietal cortices, somatosensory cortices, and mid- and dorsolateral prefrontal cortices of patients with neuropathic pain (Stern et al., 2006). These same authors argued that thalamic deactivation could be considered as the neurophysiological basis of chronic neurogenic pain. Furthermore, Sarnthein et al. hypothesized that neurogenic pain could be originated by deafferentation of excitatory inputs in the thalamus, leading to cell membrane hyperpolarization (Sarnthein et al., 2006). In this hyperpolarized state, thalamic interneurons appears to fire at a frequency range similar to the theta activity. Recent studies have shown that many RA patients reported neuropathic pain from different origins (Mendes et al., 2014; Walsh and McWilliams, 2014; Koop et al., 2015), including pain as a consequence of the use of TNF-alpha inhibitors (Birnbaum and Bingham, 2014), neurogenic inflammation (Seidel et al., 2010), and central sensitization (Meeus et al., 2012).

We also observed that RA participants and HC did not differ in delta and beta power densities at any of the ROI. A lack of statistical significance for these group differences may be attributed to a type II error in our study. At one hand, some of the previous studies observed differences on both delta and beta bands between patients with chronic pain and controls. For instance, Sarnthein et al. showed an increase in the total EEG spectrum in patients with neurogenic pain, including the delta and beta ranges (Sarnthein et al., 2006). They attributed these changes to TCD, as described above in the discussion of theta band changes. On the other hand, previous studies in patients with neuropathic chronic pain have failed to find differences between patients and HC in both delta (Bjørk et al., 2009) and beta (Vuckovic et al., 2014) EEG bands.

In this study we did not find any correlations between absolute and relative power densities and McGill scores, after controlling for depression. Correlations between pain characteristics (intensity and/or duration) and EEG power density are controversial. Schmidt et al. found a positive correlation between alpha power density and pain intensity, not at the moment of EEG evaluation but only on the one referenced in the previous 12 months (Schmidt et al., 2012). de Vries et al. only found a positive correlation between alpha peak frequency (but not power density) and pain duration, but not pain intensity (de Vries et al., 2013). On the other hand, other studies failed to find significant correlations between EEG power density and pain intensity (Jensen et al., 2007; van den Broeke et al., 2013).

Although it remains a matter of controversy, the presence of neuropathic symptoms in RA seems to be related to the presence of central sensitization, rather than a lesion of the somatosensory system itself. Since central sensitization involves the spinal cord and brain, neuropathic symptoms may be referred, even if pain is from nociceptive origin. Neuropathic pain symptoms have been identified in RA with another instrument, the PAINDetect (Koop et al., 2015; Christensen et al., 2016), which has different psychometric properties than the DN4. The DN4 has good properties to identify pain due to lesions of the somatosensory system, but most likely would not identify central sensitization adequately, as seen in Ehlers-Danlos syndrome patients (Di Stefano et al., 2016). Thus, central sensitization may have been underdiagnosed in our sample, which prevented us to identify associations between EEG variables and neuropathic pain. Future studies should use other measures of central sensitization to better classify patients and reveal if this condition has a typical EEG pattern.

As this was an initial exploratory study, the sample size did not allow us to identify whether the main findings were related to the nature of the pain or even to the use of medication to treat RA symptoms, since only two participants in the RA group were not taking medications. A third group of individuals with RA and a low level of disease activity would be required in future studies to establish a clearer relation between the observed findings and the disease itself, independent of the presence of pain.

## Conclusion

Our data suggest that subjects with RA present electroencephalographic characteristics similar to patients with chronic pain due to other etiologies. Increased absolute and relative alpha power densities at rest could be used as a general marker for the presence of chronic pain in patients with RA. This increase in alpha power density may also help to understand brain dysfunction associated with chronic pain in this population, as well as using it to develop new interventions to treat this condition.

## Author contributions

FM substantially contributed with designing the study, data acquisition, analysis and interpretation. He drafted the manuscript and helped revising it critically for important intellectual content. FQ substantially contributed with data analysis and interpretation. She drafted the manuscript and helped revising it critically for important intellectual content. PM substantially contributed with data analysis and interpretation. He helped drafting the manuscript and revising it critically for important intellectual content. JM substantially contributed with the design of the study and data analysis. He helped revising the manuscript critically for important intellectual content. SD, KS, and CL substantially contributed with the conception of the study. They helped revising the manuscript critically for important intellectual content. AB substantially contributed with conceiving and designing the study, and also with data interpretation. He drafted the manuscript and helped revising it critically for important intellectual content. All authors approved the final version to be published and agree to be fully accountable for all aspects of the work.

## Funding

This research has been supported by a grant from the Coordination for the Improvement of Higher Education Personnel (CAPES, Brazil), project A002_2013—Visiting Scholar Program. This project has also been funded by Research Support Foundation of the state of Bahia (FAPESB - Edital 028/2010).

### Conflict of interest statement

The authors declare that the research was conducted in the absence of any commercial or financial relationships that could be construed as a potential conflict of interest.

## Abbreviations

RA: rheumatoid arthritis
HC: healthy controls
HADS: Hospital Anxiety and Depression Scale
DN4: Neuropathic Pain Diagnostic Questionnaire (*Douleur Neuropathique 4*)
DAS28: Disease Activity Score in 28 Joints
ROI: Regions of Interest.
